# L1-Norm Estimation for Mixed Additive and Multiplicative Error Models: Theory and Application to GNSS Positioning

**DOI:** 10.3390/s26196042

**Published:** 2026-09-24

**Authors:** Guohong Li, Yun Shi, Shuen Wei

**Affiliations:** 1 College of Geomatics, Xi’an University of Science and Technology, Xi’an 710054, China; shiyun0908@hotmail.com; 2 School of New Energy Vehicles and Engineering, Fuzhou Technology and Business University, Fuzhou 350715, China; kuuga592@163.com

**Keywords:** mixed additive and multiplicative error models, L1-norm estimation, bias-corrected weighted least squares, Global Navigation Satellite System

## Abstract

Although Global Navigation Satellite System (GNSS) observations are verified to suffer from mixed additive and multiplicative errors, current methodologies still rely heavily on conventional additive error models (AEMs). Furthermore, existing research on mixed additive and multiplicative error models (MAMEMs) is largely confined to least squares frameworks and simulated scenarios. To address this, this study explores parameter estimation for MAMEMs using the robust L1-norm approach. Four novel algorithms are proposed: the L1-norm-based Gauss Theorem Algorithm (L1-Gauss), Linear Programming Algorithm (L1-LP), Relaxation Method (L1-RM), and Weighted Relaxation Method (L1-WRM). Among them, L1-WRM considers observation weights, while the other three algorithms treat observations as equal weights. Using real-world GNSS datasets, the proposed algorithms are compared with least squares (LS), weighted least squares (WLS) and M-robust weighted least squares (MWLS) under AEMs, as well as the bias-corrected WLS (bcWLS) estimator specifically designed for MAMEMs. Experimental results demonstrate that: Statistically significant differences exist in accuracy among the eight algorithms. In scenarios affected by multipath or non-line-of-sight (NLOS) effects, the accuracy of the bcWLS outperforms that of the WLS, and the accuracy of the L1-WRM outperforms that of the MWLS, with robust algorithms achieving superior accuracy. In scenarios without multipath or NLOS effects, the accuracy of bcWLS is virtually consistent with WLS, showing a difference in 3D Mean Error of only about 2 cm. Similarly, the accuracy of L1-WRM is virtually consistent with MWLS, with a 3D Mean Error difference of only about 8 cm; in this case, non-robust algorithms outperform robust algorithms. Regarding single-epoch execution time, the maximum computation time across the five MAMEMs algorithms is 95.5939 ms, fully satisfying the requirements for 1 Hz real-time processing. These findings validate the practical application value of the proposed algorithms in GNSS pseudorange relative positioning.

## 1. Introduction

In traditional geodetic data processing, Global Navigation Satellite System (GNSS) observations are generally assumed to be affected only by additive errors. However, with the advancement of data processing theories, researchers have demonstrated that the precision of various geodetic measurements—such as Electronic Distance Measurement (EDM), Very Long Baseline Interferometry (VLBI), Light Detection and Ranging (LiDAR), and GNSS—are influenced by both additive and multiplicative errors [1,2]. To distinguish this from the conventional additive error model, this structure is referred to as the mixed additive and multiplicative error models (MAMEMs) [1].

In the field of geodesy, Xu et al. [1] pioneered the research on the MAMEMs, deriving its ordinary least squares (LS), weighted least squares (WLS), and bias-corrected weighted least squares (bcWLS) solutions. In 2016, Shi et al. [3] investigated parameter estimation for the MAMEMs incorporating prior information. In 2021, Wang and Chen [4] proposed a virtual observation iteration solution and an A-optimal design method for the MAMEMs, effectively addressing the ill-posed nature of the design matrix. In the same year, parameter estimation for the MAMEMs under inequality constraints was explored [5]. In 2022, Wang and Chen [6] introduced the Scaled Unscented Transform for MAMEMs precision assessment, which achieves second-order accuracy and overcomes the first-order limitation of conventional formulas. In 2023, Wang Leyang proposed the Cat Swarm Optimization algorithm [7] and Artificial Gorilla Troops Optimizer [8] for parameter estimation in the MAMEMs. In 2024, Wang Leyang and Xiao Hao [9] proposed Helmert variance component estimation for the MAMEMs. To address the computational challenges associated with large-scale datasets, Wang Leyang and Peng Zhenjie [10] proposed the sequential bias-corrected weighted least squares method. In trilateration network adjustment, incorporating the parameter estimation algorithm for the MAMEMs yielded more accurate coordinate values [11]. In 2025, Wang Leyang, Hu Fangfang et al. [12] derived parameter estimation methods for MAMEMs under correlated observation and non-linear conditions [13]. These research outcomes have further promoted the development of MAMEMs in the field of geodetic data processing.

It should be noted that the aforementioned studies predominantly rely on LS criteria and simulated data. However, the LS method possesses an inherent limitation: it lacks robustness and is highly sensitive to outliers. In complex urban environments, GNSS positioning accuracy is highly susceptible to multipath and non-line-of-sight (NLOS) signals, which act as outliers and can induce errors in the order of tens of meters [14]. Compared to the LS approach, the L1-norm exhibits superior robustness against such anomalies. Consequently, this study investigates parameter estimation for the MAMEMs from the perspective of the L1-norm and validates the proposed methods using real-world GNSS datasets.

## 2. Least Squares Estimation for the MAMEMs

The MAMEMs was initially introduced by Xu et al. in 2013 [1], and is expressed as follows:

(1) $$y=f(\beta )⊙(1+\varepsilon_{m})+\varepsilon_{a}$$

where $y\in R^{n\times 1}$ denotes the observation vector; f(β) represents the function of the unknown parameters; $\beta \in R^{t\times 1}$ is the unknown parameter vector; ⊙ stands for the Hadamard product of matrices or vectors; $1\in R^{n\times 1}$ is a column vector of ones; $\varepsilon_{m}\in R^{n\times 1}$ and $\varepsilon_{a}\in R^{n\times 1}$ are the zero-mean multiplicative and additive error vectors, respectively. Furthermore, both $\varepsilon_{m}$ and $\varepsilon_{a}$ are assumed to follow a normal distribution.

If f(β) is a linear function, the MAMEMs can be reformulated as follows:

(2) $$y=(A\beta )⊙(1+\varepsilon_{m})+\varepsilon_{a}$$

Assuming that the elements within $\varepsilon_{m}$ and $\varepsilon_{a}$ are mutually independent, and that $\varepsilon_{m}$ and $\varepsilon_{a}$ are statistically independent of each other, with variances given by $\sum_{m}=I\sigma_{m}^{2}$ and $\sum_{a}=I\sigma_{a}^{2}$, respectively. According to the law of error propagation, the expectation and variance–covariance matrix of the observations can be derived as follows [1]:

(3) E(y)=Aβ

(4) $$\sum_{y}=diag\{{\left(A\beta \right)}^{2}\}\sigma_{m}^{2}+I\sigma_{a}^{2}$$

Here, $diag\{{\left(A\beta \right)}^{2}\}$ denotes a diagonal matrix with diagonal elements ${\left(A\beta \right)}^{2}$; meanwhile, $\sigma_{m}^{2}$ and $\sigma_{a}^{2}$ represent the unit weight variances of $\varepsilon_{m}$ and $\varepsilon_{a}$, respectively. In Ref. [1], three least squares solutions for the MAMEMs are provided. The standard LS solution is expressed as follows:

(5) $${\hat{\beta }}_{LS}={\left(A^{T}A\right)}^{-1}A^{T}y$$

The WLS solution is given by:

(6) $${\hat{\beta }}_{WLS}^{i+1}={\left(A^{T}{\hat{P}}_{y}^{i}A\right)}^{-1}A^{T}{\hat{P}}_{y}^{i}y+{\left(A^{T}{\hat{P}}_{y}^{i}A\right)}^{-1}\left[\begin{array}{l} {\left(y-A{\hat{\beta }}_{WLS}^{i}\right)}^{T}\frac{\partial {\hat{P}}_{y}^{i}}{\partial \beta_{1}}(y-A{\hat{\beta }}_{WLS}^{i})\\ \vdots \\ {\left(y-A{\hat{\beta }}_{WLS}^{i}\right)}^{T}\frac{\partial {\hat{P}}_{y}^{i}}{\partial \beta_{t}}(y-A{\hat{\beta }}_{WLS}^{i}) \end{array}\right]$$

where, $\frac{\partial {\hat{P}}_{y}^{i}}{\partial \beta_{i}}=\frac{-2\sigma_{m}^{2}diag(a_{i})diag(A\beta )}{{\left[diag\{{\left(A\beta \right)}^{2}\sigma_{m}^{2}\}+I\sigma_{a}^{2}\right]}^{2}}$, $a_{i}={\left[a_{1i}a_{2i}\cdots a_{ni}\right]}^{T}$ with ${\hat{P}}_{y}^{i}$ denoting the *i*-th estimate of $P_{y}$. However, the obtained WLS solution ${\hat{\beta }}_{WLS}^{i+1}$ is inherently a biased estimator [1]. By analyzing and subsequently correcting the bias of the WLS solution, an unbiased estimator is derived as follows:

(7) $${\hat{\beta }}_{bcWLS}^{i+1}={\left(A^{T}{\hat{P}}_{y}^{i}A\right)}^{-1}A^{T}{\hat{P}}_{y}^{i}y$$

Here, ${\hat{\beta }}_{bcWLS}^{i+1}$ is defined as the bcWLS solution for β, which serves as a second-order unbiased estimator. For a comprehensive derivation and further details, readers are referred to Ref. [1].

## 3. Robust L1-Norm Estimation for the MAMEMs

The MAMEMs given in Equation (2) can be equivalently reformulated as follows:

(8) $$y=(A\beta )⊙(1+\varepsilon_{m})+\varepsilon_{a}=A\beta +e$$

Here, $e=(A\beta )⊙\varepsilon_{m}+\varepsilon_{a}$ denotes the true error vector of the observations. When using the LS criterion for parameter estimation, the following objective function is usually required to be minimized:

(9) $$\min\;F_{WLS}(\hat{\beta })=V^{T}PV$$

where $P={\left(diag{\left(A\beta \right)}^{2}\sigma_{m}^{2}+\sigma_{a}^{2}\right)}^{-1}$. For the MAMEMs, due to the presence of multiplicative errors, the residual distribution exhibits heavy-tailed or asymmetric skewed characteristics. Under the LS criterion, large residuals are significantly amplified by the quadratic operation, and the influence function is unbounded. Even if a single observation is severely contaminated by outliers, its influence will be infinitely transferred to the final solution. Under the L1-norm criterion, the estimate of the unknown parameter vector is determined by minimizing the following objective function:

(10) $$\min\;F_{L1}(\hat{\beta })=\sum \left|y-A\hat{\beta }\right|$$

From a statistical perspective, the L1-norm is equivalent to assuming that the residuals follow a Laplace distribution. The Laplace distribution possesses a heavier tail than the Gaussian distribution. The L1-norm imposes a linear penalty rather than the quadratic penalty of LS on large deviations caused by outlier observations and multiplicative errors, which greatly reduces the impact of extreme residuals on the overall parameter estimation. Moreover, the influence function of the L1-norm estimation is bounded; when a certain observation residual increases, its derivative at the extremum point depends solely on the sign (±1) and does not increase infinitely with the magnitude of the residual. This ensures that magnitude fluctuations caused by multiplicative errors in the MAMEMs do not dominate the optimization direction. When considering observation weights, the objective function of the weighted L1-norm is:

(11) $$\min\;F_{WL1}(\hat{\beta })=\sum \sqrt{P}\left|y-A\hat{\beta }\right|=\frac{\left|V\right|}{\sqrt{diag{\left(A\beta \right)}^{2}\sigma_{m}^{2}+\sigma_{a}^{2}}}$$

Although the bcWLS solution under the LS criterion also considers the variance information of multiplicative and additive errors, the residual vector during the estimation of these components is still driven by the LS solution. If the initial weights are inappropriate, it easily leads to deviations in the estimated variance components, inducing instability in the convergence of the stochastic model. In contrast, the weighted L1-norm rapidly provides a residual vector unperturbed by outlier and multiplicative errors in the early stage of iteration, rendering the subsequent variance components more objective, which in turn feeds back into the stochastic model, forming a virtuous cycle of robust estimation and accurate weighting.

From a historical perspective, the inception of the L1-norm criterion predates that of the least squares concept. According to historical records, between 1755 and 1757, the mathematician Boscovich employed the L1-norm concept for linear fitting research, approximately 40 years prior to the introduction of the least squares method by Gauss. However, owing to its overwhelming computational complexity, the application of this criterion remained dormant for a prolonged period [15]. It was not until 1987, when Koenker and D’Orey [16] introduced linear programming to resolve its computational bottlenecks, that the L1-norm criterion began to garner increasing attention in practical applications.

### 3.1. Gauss Theorem Algorithm

Least squares estimation relies on the criterion of minimizing the weighted sum of squared residuals. Its objective function is a smooth and differentiable quadratic convex function, which allows for the derivation of a unique analytical solution by setting its derivative to zero. In contrast, the objective function of the L1-norm criterion is an absolute value function (Equation (10)). It is characterized by being non-smooth and non-differentiable at the origin, thereby precluding the derivation of an analytical solution through conventional differentiation. In 1809, Gauss demonstrated that a regression line fitted under the L1-norm criterion inevitably passes through at least two sample points, a theorem that can be generalized to scenarios involving *P* independent variables [15]. Consequently, when applying the Gauss Theorem Algorithm to obtain the L1-norm solution for the MAMEMs (assuming equal weights), the principal procedure encompasses the following steps:

Step1. Let *n* represent the total number of observations, and *k* represent the required number of observations. Generate $C_{n}^{k}$ combinations of samples.

Step2. Calculate the estimation ${\hat{\beta }}_{i}=X_{i}^{-1}y_{i}$ for each combination, as well as sum of the absolute residuals $v_{{\hat{\beta }}_{i}}=\sum_{t=1}^{n}\left|v_{t}\right|$.

Step3. Identify the ${\hat{\beta }}_{i}$ corresponding to the smallest value of sum of the absolute residuals $v_{{\hat{\beta }}_{i}}$, which represents the theoretical estimation of the least absolute deviation.

As can be seen from the computational steps, although Gauss theorem method can yield theoretical estimates, its computational burden becomes extremely massive when the data volume is large, making it potentially unfeasible to implement. For instance, selecting three samples out of 300 observations requires 14,350,100 computations; when facing millions or even hundreds of millions of data points, obtaining theoretical estimates using this method is virtually impossible. For GNSS pseudorange relative positioning, what is processed are the pseudorange signals of various satellite constellations acquired by the receiver. Currently, the mainstream satellite systems mainly include GPS, BDS, GLONASS and Galileo. In open-sky environments, the number of visible satellites in mid-latitude regions is basically around 30 (cut-off elevation angle 15°). Assuming that the triple-frequency information broadcast by each satellite can be acquired, the number of Double-differenced pseudorange observations is approximately 78, requiring the processing of 76,076 combinations, which is completely manageable for current receivers.

### 3.2. Linear Programming Algorithm

The introduction of linear programming effectively resolved the computational burden associated with the L1-norm criterion. Charnes et al. [17] demonstrated that the minimization of $\sum_{i=1}^{n}\left|y_{i}-a-b^{T}x_{i}\right|$ can be equivalently transformed into a linear programming problem:

First, let $c={\left[a\;b\right]}^{T}Z_{i}={\left[1\;x_{i}\right]}^{T},i=1,\cdots n$, and transform the objective function into:

(12) $$T(c)=\sum_{i=1}^{n}\left|y_{i}-a-b^{T}x_{i}\right|=\sum_{i=1}^{n}\left|y_{i}-c^{T}Z_{i}\right|$$

An arbitrary vector *c* can be expressed in the form *d*1 − *d*2 with each component of *d*1, *d*2 non-negative, i.e., *d*1 ≥ 0, *d*2 ≥ 0. Denoting the matrices *A* and *B*:

$$A={\left[Z_{1}^{T}\cdots Z_{n}^{T}\right]}^{T},B=\left[\begin{array}{l} I_{n}\;\;A\;\;-A\\ I_{n}\;\;-A\;\;A \end{array}\right]$$

$$y={\left[y_{1}\cdots y_{n}\right]}^{T}\;\;r={\left[r_{1}\cdots r_{n}\right]}^{T}\;\;l={\left[1\cdots 1\right]}^{T}\;\;O={\left[0\cdots 0\right]}^{T}$$

where $I_{n}$ is a unit matrix of order *n*. l and O are n-dimensional, a linear programming problem is introduced: minimize the objective function (Equation (14)) subject to the constraints (Equation (13)).

(13) $$B{\left[r\;d_{1}\;d_{2}\right]}^{T}\geq \left[y-y\right],\left[r\;d_{1}\;d_{2}\right]\geq 0$$

(14) $$\left[l^{T},O^{T},O^{T}\right]{\left[r\;d_{1}\;d_{2}\right]}^{T}=\sum_{i=1}^{n}r_{i}$$

Upon obtaining the optimal solution $r,d_{1},d_{2}$ for the linear programming problem, the substitution *c* = *d*1 − *d*2 minimizes the objective function, yielding a minimum value of $\sum_{i=1}^{n}r_{i}$. Regarding the methods for solving linear programming problems, the main approaches include the simplex method, interior-point method, and dual method, among which the most basic and commonly used is the simplex method. This algorithm was proposed by American mathematician George Dantzig in 1947 [18], and complete programs are currently available for direct use. Consequently, this study employs this method to perform L1-norm estimation for the MAMEMs.

The mathematical model for a linear programming problem is formulated as follows:

(15) f(β)=Cβ=min

Subject to the constraints:

(16) Aβ=bβ≥0

In this context, the value of β is referred to as a solution to the linear programming problem. A solution that satisfies the constraint conditions is called a feasible solution. There can be multiple feasible solutions, and the goal of linear programming is to find the one that minimizes the objective function, known as the optimal solution. The computational steps of the simplex method are as follows [19]:

Step 1. Formulate the residual equations:

(17) $$V-A\hat{\beta }=-y$$

Linear programming requires all unknown parameters to be non-negative. Let:

$$v_{i}=\mu_{i}-\omega_{i},\;\mu_{i}\geq 0,\omega_{i}\geq 0,\;i=1,2,3\cdots n$$

$$\beta_{j}=\alpha_{j}-\theta_{j},\;\alpha_{j}\geq 0,\alpha_{j}\geq 0,\;j=1,2,\cdots t$$

Equations (15) and (16) can be reformulated as:

(18) $$[B\;N]\left[\begin{array}{l} \beta_{B}\\ \beta_{N} \end{array}\right]=b$$

(19) $$[C_{B}\;C_{N}]\left[\begin{array}{l} \beta_{B}\\ \beta_{N} \end{array}\right]=\min$$

where

$$\begin{array}{l} B=I;\;N=[-A\;\;A\;\;-I];\;b=[-y_{1}\cdots -y_{n}]\\ C_{B}=[1_{1}\cdots 1_{n}];\;\;C_{N}=[0_{1}\cdots 0_{t}\;\;0_{1}\cdots 0_{t}\;\;1_{1}\cdots 1_{n}];\\ \beta_{B}={\left[\mu_{1}\cdots \mu_{n}\right]}^{T};\;\;\beta_{N}={\left[\alpha_{1}\cdots \alpha_{t}\;\;\theta_{1}\cdots \theta_{t}\;\;\omega_{1}\cdots \omega_{t}\right]}^{T} \end{array}$$

Let the initial Simplex Tableau be denoted by S: $S=\left(\begin{array}{ccc} \beta_{B}^{T} & \beta_{N}^{T} & 0\\ B & N & b\\ C_{B} & C_{N} & 0 \end{array}\right)$

Step 2. Ensure that all constant terms in the residual equations are non-negative:

If the constant term $-y_{k}$ of the *k*-th residual equation is less than 0, multiply both sides of the equation by −1 to ensure non-negativity. Simultaneously, swap the column $\mu_{k}$ in matrix B with the column $\omega_{k}$ in matrix N. Likewise, swap the corresponding columns in $C_{B}$ and $C_{N}$, as well as in $\beta_{B}$ and $\beta_{N}$, to obtain the new matrices $B^{0}$, $N^{0}$, $C_{B}^{0}$, $C_{N}^{0}$, $\beta_{B}^{0}$, $\beta_{N}^{0}$.

Step 3. Solve for the basic feasible solution:

Set the non-basic parameters $\beta_{N}^{0}=0$, then $\beta_{B}^{0}={\left(B^{0}\right)}^{-1}b$. If any value in $\beta_{B}^{0}$ is negative, it indicates that the simplex method has no solution, and the computation stops. Otherwise, the basic feasible solution is:

(20) $$\beta^{0}=\left[\begin{array}{l} \beta_{B}^{0}\\ \beta_{N}^{0} \end{array}\right]=\left[\begin{array}{l} {\left(B^{0}\right)}^{-1}b\\ 0 \end{array}\right]$$

Step 4. Calculate the reduced costs:

Calculate the reduced costs $r^{0}=C_{N}^{0}-C_{B}^{0}{\left(B^{0}\right)}^{-1}N^{0}$. If all values are greater than or equal to 0, it indicates that the optimal solution has been achieved; proceed to Step 8.

Step 5. Determine the entering basis column:

Among all $r^{0}<0$, find the value with the maximum absolute value, denoted as $r_{j}^{0}$. The corresponding *j*-th column of $N^{0}$ is selected as the entering basis column.

Step 6. Determine the leaving basis column:

Calculate the ratio of ${\left(B^{0}\right)}^{-1}N_{j}^{0}$ to ${\left(B^{0}\right)}^{-1}b$, denoted as $s^{0}$. Among all $s^{0}>0$, find the minimum positive value, denoted as $s_{m}^{0}$. The corresponding *m*-th row of $B^{0}$ is selected as the leaving basis column.

Step 7. Iterative computation:

Swap the *m*-th column of $B^{0}$ with the *j*-th column of $N^{0}$. Similarly, swap the m-th column of $C_{B}^{0}$ with the *j*-th column of $C_{N}^{0}$, and the *m*-th column of $\beta_{B}^{0}$ with the *j*-th column of $\beta_{N}^{0}$. This yields the updated matrices $B^{1}$, $N^{1}$, $C_{B}^{1}$, $C_{N}^{1}$, $\beta_{B}^{1}$, $\beta_{N}^{1}$. Return to Step 3.

Step 8. Final computation:

Using the obtained optimal solution $\beta_{B}^{i}$, $\beta_{N}^{i}$, calculate the final residuals V and the parameter estimates $\hat{\beta }$.

It should be noted that Equations (12)–(14) represent the standard linear programming in matrix form for the equal-weight L1-norm solution, whereas Equations (15)–(20) outline the specific implementation steps of the simplex algorithm; mathematically, the two are completely equivalent.

### 3.3. Relaxation Method

The objective function of the L1-norm criterion can be formulated as follows:

(21) $$F_{L1}(\hat{\beta })=\min\sum_{i=1}^{n}\left|v_{i}\right|$$

When weighted least squares is utilized, the objective function is:

(22) $$F_{WLS}(\hat{\beta })=\min\sum_{i=1}^{n}\left(\omega_{i}v_{i}^{2}\right)$$

Here $\omega_{i}$ denotes the weight of observation $y_{i}$. Comparison of Equations (21) and (22) shows that when $v_{i}\neq 0$, the following equation holds.

(23) $$\left|v_{i}\right|=\frac{1}{\left|v_{i}\right|}v_{i}^{2}$$

Equation (21) can be written as:

(24) $$F_{L1}(\hat{\beta })=\min\sum_{i=1}^{n}\left|v_{i}\right|=\min\sum_{i=1}^{n}\left(\frac{1}{\left|v_{i}\right|}v_{i}^{2}\right)$$

Let $\omega_{i}=1/\left|v_{i}\right|$, Equation (24) can be rewritten as:

(25) $$F_{L1}(\hat{\beta })=\min\sum_{i=1}^{n}\left(\omega_{i}v_{i}^{2}\right)$$

Comparison of Equations (21) and (25) shows that when $\omega_{i}=1/\left|v_{i}\right|$ and $v_{i}\neq 0$, the following equation holds.

(26) $$F_{WLS}(\hat{\beta })=F_{L1}(\hat{\beta })$$

Consequently, the L1-norm estimates can be derived by adopting the framework of the weighted least squares method. Since residual information is required, an iteratively reweighted approximation approach is usually adopted to obtain the estimates. In practical computation, when the residual $v_{i}$ approaches zero, the weight $\omega_{i}$ tends toward infinity, which easily causes numerical oscillations or convergence to local minima. To avoid this phenomenon, a smoothing factor $\delta =10^{-6}$ is introduced, updating the weight as:

(27) $$\omega_{i}=\frac{1}{\sqrt{v_{i}^{2}+\delta^{2}}}$$

At this point, the objective function is transformed into a smooth convex function. According to the convergence theories of Coleman [20] and Daubechies [21], the first-order smoothed iteratively reweighted least squares strictly satisfies the monotonic decrease in the objective function under any data configuration and achieves global convergence to the δ global optimal solution. The calculation steps are as follows:

Step 1. Apply least squares to compute the residuals $v_{i}^{\left(0\right)}$.

Step 2. In the *k*-th iteration, calculate the smoothed weight for each observation: $\omega_{i}^{k}=\frac{1}{\sqrt{v_{i}^{2(k)}+\delta^{2}}}$.

Step 3. Update the parameter estimates and residuals via least squares.

Step 4. Check algorithm convergence. Stop the iteration and output the final solution when the triple termination criteria are satisfied; otherwise, return to Step 2 for the next iteration. The termination criteria are $\left\|{\hat{\beta }}_{L1}^{i+1}-{\hat{\beta }}_{L1}^{i}\right\|\leq 10^{-6}$. $\frac{\left|F_{L1}{\left(\hat{\beta }\right)}^{k+1}-F_{L1}{\left(\hat{\beta }\right)}^{k}\right|}{F_{L1}{\left(\hat{\beta }\right)}^{k}}\leq 10^{-6}$, $\left\|A^{T}\mathrm{sgn}(v^{k+1})\right\|\leq 10^{-6}$.

### 3.4. Weighted Relaxation Method

The above-mentioned Gauss theorem method, linear programming, and relaxation factor method all treat the observations as equal precision. For the MAMEMs, however, the observations themselves are of unequal precision. The observation weight $p_{i}={\left({\left(a_{i}\beta \right)}^{2}\sigma_{m}^{2}+\sigma_{a}^{2}\right)}^{-1}$ is not only related to the unknown parameters β, but also to the variance information of both the multiplicative and additive errors. Therefore, the observation weighting issue must be taken into consideration.

For the MAMEMs, the variance of the residual $v_{i}$ is given by:

(28) $$\sigma_{v_{i}}^{2}={\left(a_{i}\beta \right)}^{2}\sigma_{m}^{2}+\sigma_{a}^{2}$$

Then, the objective function of the weighted L1-norm estimation that incorporates observation weights is expressed as:

(29) $$\sum_{i=1}^{n}\frac{\left|v_{i}\right|}{\sqrt{{\left(a_{i}\beta \right)}^{2}\sigma_{m}^{2}+\sigma_{a}^{2}}}=\min$$

By introducing a dual reweighting factor, the diagonal elements of the equivalent diagonal weight matrix ${\hat{P}}^{k}=diag({\hat{p}}_{1}^{k},\ldots ,{\hat{p}}_{n}^{k})$ are decomposed into two components. The first component is the variance component weighting factor:

(30) $$\omega_{i,vce}^{k}=\frac{1}{\sqrt{{\left(a_{i}{\hat{\beta }}^{k}\right)}^{2}{\hat{\sigma }}_{m}^{2,k}+{\hat{\sigma }}_{a}^{2,k}}}$$

The second component is the L1 robust weighting factor:

(31) $$\omega_{i,L1}^{k}=1/\sqrt{v_{i}^{2(k)}+\delta^{2}}$$

The expression for the composite equivalent weight is:

(32) $${\hat{p}}_{i}^{k}=\omega_{i,vce}^{k}\cdot \omega_{i,L1}^{k}=\frac{1}{\sqrt{{\left(a_{i}{\hat{\beta }}^{k}\right)}^{2}{\hat{\sigma }}_{m}^{2,k}+{\hat{\sigma }}_{a}^{2,k}}\cdot \sqrt{v_{i}^{2(k)}+\delta^{2}}}$$

Under this weight matrix, the parameter estimates for the weighted L1-norm estimation are given by:

(33) $${\hat{\beta }}^{k}={\left(A^{T}{\hat{P}}^{k}A\right)}^{-1}A^{T}{\hat{P}}^{k}y$$

Since the equivalent weight contains the variance information of both the multiplicative and additive errors, a variance component estimation algorithm is required to obtain these estimates from the residuals.

Expanding the MAMEMs element by element, it can be expressed as:

(34) $$y_{i}=\mu_{i}⊙(1+\varepsilon_{m,i})+\varepsilon_{a,i}$$

where $\mu_{i}=a_{i}^{T}\beta$ represents the true value of the observation, and the true error of the observation can be expressed as:

(35) $$e=y-(A\beta )=\mu ⊙\varepsilon_{m}+\varepsilon_{a}$$

For the MAMEMs, it is generally assumed that the additive error follows a normal distribution $\varepsilon_{a}∼N(0,\sigma_{a}^{2}Q_{a})$ and the multiplicative error follows a normal distribution $\varepsilon_{m}∼N(0,\sigma_{m}^{2}Q_{m})$ with no correlation between the two errors, i.e., $Cov(\varepsilon_{m},\varepsilon_{a})=0$. According to the law of variance–covariance propagation, the variance structure of the true error can be derived as follows:

(36) $$D(e)=E[(\mu ⊙\varepsilon_{m}+\varepsilon_{a}){\left(\mu ⊙\varepsilon_{m}+\varepsilon_{a}\right)}^{T}]=\mu \mu^{T}⊙\sigma_{m}^{2}Q_{m}+\sigma_{a}^{2}Q_{a}$$

To facilitate subsequent derivations, the cofactor base matrices of the multiplicative error and additive error are defined as:

(37) $$\left\{\begin{array}{l} Z_{m}=\mu \mu^{T}⊙Q_{m}\\ Z_{a}=Q_{a} \end{array}\right.$$

The variance of the true error can be expressed in the following structure:

(38) $$D(e)=\sigma_{m}^{2}Z_{m}+\sigma_{a}^{2}Z_{a}$$

Since $Z_{m}$ depends on the true values of the unknown parameters, an iterative method must be used during the solution process. For the MAMEMs, the bcWLS method is usually adopted because this solution belongs to a second-order unbiased estimation. According to Xu’s research findings [3], the relationship between the bias-corrected solution of the mixed additive and multiplicative error model and the true parameters is as follows:

(39) $${\hat{\beta }}_{bc}=\beta +N^{-1}A^{T}\sum_{y}^{-1}e+b_{c\beta }$$

where $N=A^{T}\sum_{y}^{-1}A$ is the normal equation matrix, and $b_{c\beta }$ denotes the inherent second-order bias term of the WLS solution, originating from the coupling between the covariance matrix and unknown parameters caused by multiplicative errors, which can be obtained by:

(40) $$b_{c\beta }=N^{-1}A^{T}\sum_{y}^{-1}(D_{ab}\sum_{m}D_{a\beta }+D_{a\beta }\sum_{m}D_{ab})\sum_{y}^{-1}(I_{n}-AN^{-1}A^{T}\sum_{y}^{-1})e$$

where $D_{ab}$ is a diagonal matrix with elements $a_{i}B\varepsilon_{ma}$, B is a matrix to be determined that is independent of $\varepsilon_{ma}$, $\varepsilon_{ma}={\left(\varepsilon_{m}^{T},\varepsilon_{a}^{T}\right)}^{T}$, $D_{a\beta }=diag(a_{i}\beta )$. The corresponding residuals for the bcWLS solution are:

(41) $$\begin{array}{ll} V & =A{\hat{\beta }}_{bc}-y=A\beta +AN^{-1}A^{T}\sum_{y}^{-1}e+Ab_{c\beta }-A\beta -e\\ & =AN^{-1}A^{T}\sum_{y}^{-1}e-e+Ab_{c\beta }\\ & =(AN^{-1}A^{T}\sum_{y}^{-1}-I_{n})e+Ab_{c\beta }\\ & =Je+A\psi e\\ & =(J+A\psi )e\\ & =Fe \end{array}$$

where $J=AN^{-1}A^{T}\sum_{y}^{-1}-I_{n}$ is the projection matrix and ψ is the coefficient matrix obtained by rearranging the second-order bias term, with its specific form given by:

(42) $$\psi =N^{-1}A^{T}\sum_{y}^{-1}(D_{ab}\sum_{m}D_{a\beta }+D_{a\beta }\sum_{m}D_{ab})\sum_{y}^{-1}(I_{n}-AN^{-1}A^{T}\sum_{y}^{-1})$$

According to the law of error propagation, the variance–covariance matrix of the residuals is obtained as:

(43) $$D(V)=FD(e)F^{T}=\sigma_{m}^{2}M_{m}+\sigma_{a}^{2}M_{a}$$

where $M_{m}=FZ_{m}F^{T}$, $M_{a}=FZ_{a}F^{T}$. Following the idea of Helmert variance component estimation [22,23], for the MAMEMs, the quadratic forms of residuals are constructed using the defined cofactor matrices of multiplicative and additive errors:

(44) $$\left\{\begin{array}{l} \Omega_{m}=V^{T}Z_{m}^{-1}V\\ \Omega_{a}=V^{T}Z_{a}^{-1}V \end{array}\right.$$

Based on the trace formula for the expectation of quadratic forms, the expectations of Equation (44) read:

(45) $$\left\{\begin{array}{l} E(\Omega_{m})=tr(Z_{m}^{-1}D(V))\\ E(\Omega_{a})=tr(Z_{a}^{-1}D(V)) \end{array}\right.$$

Substituting the residual variance into the above expressions and expanding yields:

(46) $$\left\{\begin{array}{l} E(\Omega_{m})=tr(Z_{m}^{-1}D(V))=\sigma_{m}^{2}tr(Z_{m}^{-1}M_{m})+\sigma_{a}^{2}tr(Z_{m}^{-1}M_{a})\\ E(\Omega_{a})=tr(Z_{a}^{-1}D(V))=\sigma_{m}^{2}tr(Z_{a}^{-1}M_{m})+\sigma_{a}^{2}tr(Z_{a}^{-1}M_{a}) \end{array}\right.$$

Define the coefficients of the system of equations as:

(47) $$\left\{\begin{array}{l} S_{11}=tr(Z_{m}^{-1}M_{m})\;S_{12}=tr(Z_{m}^{-1}M_{a})\\ S_{21}=tr(Z_{a}^{-1}M_{m})\;S_{22}=tr(Z_{a}^{-1}M_{a}) \end{array}\right.$$

Referring to the framework of Helmert variance component estimation [22,23], the mathematical expectations in Equation (46) are replaced by residuals derived from adjustment during computation. Accordingly, the following bivariate linear system of equations is established:

(48) $$\left\{\begin{array}{l} S_{11}{\hat{\sigma }}_{m}^{2}+S_{12}{\hat{\sigma }}_{a}^{2}=V^{T}Z_{m}^{-1}V\\ S_{21}{\hat{\sigma }}_{m}^{2}+S_{22}{\hat{\sigma }}_{a}^{2}=V^{T}Z_{a}^{-1}V \end{array}\right.$$

Rewriting it into matrix form yields:

(49) $$S\hat{\theta }=W_{\theta }$$

where $\hat{\theta }={\left[{\hat{\sigma }}_{m}^{2}\;{\hat{\sigma }}_{a}^{2}\right]}^{T}\;W_{\theta }={\left[V^{T}Z_{m}^{-1}V\;V^{T}Z_{a}^{-1}V\right]}^{T}$, $S=\left(\begin{array}{cc} tr(Z_{m}^{-1}M_{m}) & tr(Z_{m}^{-1}M_{a})\\ tr(Z_{a}^{-1}M_{m}) & tr(Z_{a}^{-1}M_{a}) \end{array}\right)$

Since the number of variance components is equal to the number of equations, Equation (49) has a unique solution:

(50) $$\hat{\theta }=S^{-1}W_{\theta }$$

After the variance information of multiplicative and additive errors is acquired via the above variance component estimation, the weights of observations are only dependent on the unknown parameters. The final parameter estimates can be obtained by iteratively updating the unknown parameters.

To rigorously account for the varying weights of the observations, the procedure for the Weighted Relaxation Method is outlined as follows:

Step 1. Obtain the initial residuals $v_{i}^{\left(0\right)}$ using least squares estimation.

Step 2. In the k-th iteration, calculate the smoothed weight for each observation: $\omega_{i}^{k}=\frac{1}{\sqrt{v_{i}^{2(k)}+\delta^{2}}}$, and obtain the parameter estimates.

Step 3. Set the initial prior values of the variance components ${\hat{\sigma }}_{a}^{2,0}$ and ${\hat{\sigma }}_{m}^{2,0}$, and construct the variance component weighting factor using the parameter estimates obtained in Step 2.

Step 4. Construct the equivalent weight matrix, apply weighted least squares to obtain the parameter estimates and residuals, and perform variance component estimation using the residuals to update the variances of the multiplicative and additive errors.

Step 5. Check algorithm convergence. Stop the iteration and output the final solution when the quadruple termination criteria are satisfied; otherwise, return to Step 2 for the next iteration. The termination criteria are $\left\|{\hat{\beta }}_{L1}^{i+1}-{\hat{\beta }}_{L1}^{i}\right\|\leq 10^{-6}$, $\frac{\left|F_{L1}{\left(\hat{\beta }\right)}^{k+1}-F_{L1}{\left(\hat{\beta }\right)}^{k}\right|}{F_{L1}{\left(\hat{\beta }\right)}^{k}}\leq 10^{-6}$, $\left\|A^{T}\mathrm{sgn}(v^{k+1})\right\|\leq 10^{-6}$, $\left\|{\hat{\theta }}^{k+1}-{\hat{\theta }}^{k}\right\|\leq 10^{-6}$.

## 4. Examples and Analysis

To verify the feasibility and value of the proposed algorithm, experimental validation is conducted using real-world GNSS data. Double-differenced pseudorange is a commonly used observable in GNSS relative pseudorange positioning, as differencing can effectively mitigate the effects of receiver and satellite clock errors, as well as atmospheric delays. The Double-differenced pseudorange observation equation is given by:

(51) $$\nabla \Delta P_{AB}^{ij}=\nabla \Delta \rho_{AB}^{ij}+\nabla \Delta V_{ionAB}^{ij}+\nabla \Delta V_{tropAB}^{ij}+\nabla \Delta M_{ulAB}^{ij}+\nabla \Delta \varepsilon_{AB}^{ij}$$

where ∇Δ denotes the double-difference operator between stations and satellites; $\nabla \Delta P_{AB}^{ij}$ represents the Double-differenced pseudorange observable; $\nabla \Delta \rho_{AB}^{ij}$ is the geometric distance difference between receivers A, B and satellites *i*, *j*; $\nabla \Delta V_{ionAB}^{ij},\nabla \Delta V_{tropAB}^{ij}$ represent the Double-differenced ionospheric and tropospheric errors, respectively; $\nabla \Delta M_{ulAB}^{ij}$ denotes the Double-differenced multipath error; and $\nabla \Delta \varepsilon_{AB}^{ij}$ represents other pseudorange measurement noise. The linearized form of Equation (51) is:

(52) $$V_{P}=B_{P}X_{P}-L_{P}$$

where $V_{P}$ is the vector of observation corrections; $X_{P}$ is the vector of approximate coordinate corrections to be estimated, $X_{P}=[\delta_{x}\delta_{y}\delta_{z}]$; $B_{P}$ is the design matrix; and $L_{P}$ is the Double-differenced pseudorange residual vector, expressed as:

(53) $$L_{P}=\nabla \Delta P_{AB}^{ij}-\nabla \Delta \rho_{AB}^{ij}-\nabla \Delta V_{ionAB}^{ij}-\nabla \Delta V_{tropAB}^{ij}-\nabla \Delta M_{ulAB}^{ij}-\nabla \Delta \varepsilon_{AB}^{ij}$$

Assuming the observation weight is P, the coordinate corrections can be obtained via weighted least squares (WLS):

(54) $$X_{P}={\left(B_{p}^{T}PB_{P}\right)}^{-1}B_{p}^{T}PL_{P}$$

In conventional GNSS positioning, weight functions primarily include the sine function model and the exponential function model [24,25]. While the parameters of the exponential model rely heavily on empirical values, the sine model determines weights based on the elevation angles of the reference and rover satellites. Due to its broader applicability, the sine model’s weight calculation is expressed as:

(55) $$P=\sin E_{R}/\sin E_{S}$$

where $E_{R}$ is the elevation angle of the reference satellite, and $E_{S}$ is the elevation angle of the observed satellite.

According to the principles of GNSS positioning, the ionospheric and tropospheric errors in pseudorange observations are directly related to the path length of the satellite signal passing through the atmosphere (elevation angle). When a satellite is near the zenith (high elevation angle), the signal path through the atmosphere is the shortest, resulting in smaller delays. Conversely, when a satellite is near the horizon, the signal must traverse a thicker layer of the atmosphere, causing the delay to increase several-fold (typically proportional to the mapping function 1/sinE). For instance, the tropospheric error in the zenith direction is usually 2 m to 3 m; whereas, near the horizon (elevation angle of 10°) the tropospheric error can exceed 20 m [26]. Consequently, in conventional data processing, observation weights are commonly obtained according to Equation (55). As shown in the above analysis, satellites at different elevation angles correspond to different errors—that is, different variances of random errors—exhibiting clear multiplicative characteristics. Even though double-differencing can reduce the impact of ionospheric and tropospheric errors, it cannot alter the multiplicative nature of the residual error variance varying with the path length.

Based on the above analysis, eight algorithms are employed in this paper for data processing, as listed in Table 1. The LS, WLS, and MWLS algorithms treat observation errors as additive errors, whereas the five algorithms—bcWLS, L1-Gauss, L1-LP, L1-RM, and L1-WRM—treat observation errors as mixed additive and multiplicative errors. Among them, WLS performs weighted least squares under the AEMs, with its weights obtained from Equation (55); MWLS executes robust weighted least squares under the AEM, with its weights jointly formed by Equation (55) and the IGGIII weight factor $k_{1}=1.5,k_{2}=3.0$. The bcWLS performs bias-corrected weighted least squares under the MAMEMs, with its weights obtained via variance component estimation according to Equations (39)–(49). L1-Gauss performs combinatorial optimization, searching through all possible combinations to obtain the optimal solution. L1-LP executes the simplex method to search for the optimal solution. L1-RM performs iteratively reweighted least squares, with weights obtained from Equation (27). L1-WRM executes iteratively reweighted least squares under the mixed additive–multiplicative error model, with weights obtained from Equation (32). Note: L1-RM merely utilizes the concept of weighted least squares to derive the L1-norm solution, without considering the intrinsic observation weights themselves.

Traditional GNSS data processing theory is based on the AEMs. To better analyze the positioning accuracy under the MAMEMs, the impacts of the observation environment and baseline length were taken into account during data collection. In Scenario 1, the rover was set up between academic buildings (Figure 1a), where the heights of the buildings to the south and north were approximately 20 m, the corridor height to the east was about 15 m, and the field of view to the west was open and unobstructed. The distance between the base station and the rover was around 200 m. The data sampling rate was 1 Hz, totaling 5694 s. In Scenario 2, the rover was set up on a balcony of an academic building (Figure 1b), with the buildings to the east and north being approximately 15 m high, while the west and south directions were unobstructed. The data sampling rate was 1 Hz, totaling 15270 s. The distance between the base station and the rover was around 100 m. In Scenario 3, the rover was set up on a pedestrian bridge in a park (Figure 1c), featuring an open surrounding environment without obstructions. The data sampling rate was 1 Hz, totaling 2146 s. The distance between the base station and the rover was around 6 km. In Scenario 4, the rover was set up on a school rooftop (Figure 1d), with an open surrounding environment and no obstructions. The data sampling rate was 1 Hz, totaling 18,630 s. The distance between the base station and the rover was around 42 km. SOUTH Galaxy G1 receivers with S82X_T970A antenna (SOUTH SURVEYING & MAPPING TECHNOLOGY CO., LTD., Guangzhou, China) types were used for Scenarios 1 and 2. Hi-Target TS7 receivers with HITTS7 antenna (Guangzhou Hi-Target Navigation Tech Co., Ltd., Guangzhou, China) types were used for Scenarios 3 and 4. The cut-off elevation angle for all four scenarios was set to 15°.

After data collection, the Trimble Business Center (TBC v5.90) was used to process the baselines and obtain the rover coordinates, which were taken as the reference true values (WGS84). Only the first frequency band information of BDS and GPS satellites was utilized in the processing. Exactly 11 satellites were involved in the processing for Scenario 1, 13 satellites for Scenario 2, 25 satellites for Scenario 3, and 16 satellites for Scenario 4. The Double-differenced pseudorange residuals for the four scenarios are shown in Figure 2.

As shown in Figure 2, both Scenario 1 and Scenario 2 were affected by multipath or NLOS effects, resulting in Double-differenced pseudorange residuals exceeding 20 m. In Scenario 3, the fluctuation range of Double-differenced pseudorange residuals was controlled within ±5 m, and in Scenario 4, it was controlled within ±3 m. To better analyze and compare the accuracy differences among the eight algorithms, the relationship between accuracy and baseline length, and whether the runtime of each algorithm can meet the real-time processing requirements of GNSS positioning, a Kruskal–Wallis overall test [27] was performed on the positioning accuracy of the eight algorithms to determine whether significant differences exist. Concurrently, a Wilcoxon Signed-Rank Test [28] was conducted between each algorithm and L1-WRM to verify whether the accuracy of the L1-WRM algorithm is significantly superior to that of the other algorithms. All algorithms were executed in epoch-by-epoch independent solution mode. The positioning error for each epoch was obtained by differencing the estimated 3D rover coordinates from the reference true coordinates. The positioning accuracy, significance test results, and runtimes of the eight algorithms under Scenario 1 are summarized in Table 2 and Table 3. Software and configuration: MATLAB 2022b, CPU: Intel (R) Core (TM) i9-9980XE CPU@3.00GHz 3.00GHz, GPU: NVIDIA GeForce RTX 2080 Ti.

As can be seen from Table 2, among the three algorithms based on the AEMs, the MWLS achieves the highest accuracy, which adopts the IGGIII robust weight factor. Comparing the MWLS with the four L1-norm algorithms under the MAMEMs reveals that all four L1-norm algorithms outperform MWLS in accuracy. Among the four L1-norm algorithms, L1-Gauss (with 84 combinations) yields consistent accuracy with L1-LP, while L1-WRM, which accounts for observation weights, achieves the highest accuracy. Comparing WLS with bcWLS shows that bcWLS, based on the MAMEMs, offers superior accuracy. The main reason is that the weights in WLS are fixed and cannot resist outlier interference, whereas the weights in bcWLS are iterative weights that adjust appropriately with observations, thereby providing a certain level of robustness. As shown in Table 3, both the overall test significance results (p≪0.001) and the paired test results (p≪0.001) statistically confirm that the performance advantage of L1-WRM is not due to random fluctuations. Its 95% Bootstrap confidence interval ([1.9833, 2.0549] m) shows complete separation without overlap compared to other algorithms, further verifying the high robustness and reliability of this method. Furthermore, the maximum single-epoch runtime among all eight algorithms is only 95.5939 ms (L1-LP), fully meeting the engineering requirements for 1 Hz real-time processing.

Scenario 2, like Scenario 1, also contains multipath or NLOS effects. Analyzing the condition of Scenario 2, the positioning accuracy of the eight algorithms is shown in Table 4 and Table 5:

As can be seen from Table 4, among the three algorithms under the AEMs, the MWLS algorithm achieves the highest accuracy. Comparing the MWLS with the four L1-norm algorithms under the MAMEMs reveals that the three L1-norm algorithms without considering weights are inferior to the MWLS algorithm in accuracy; whereas, L1-WRM, which incorporates observation weights, outperforms the MWLS algorithm. Among the four L1-norm algorithms, L1-Gauss (with 165 combinations) yields consistent accuracy with L1-LP, while L1-WRM, which accounts for observation weights, achieves the highest accuracy. Comparing WLS with bcWLS shows that bcWLS, based on the MAMEMs, offers superior accuracy. As shown in Table 5, both the overall test significance results (p≪0.001) and the paired test results (p≪0.001) statistically confirm that the performance advantage of L1-WRM is not due to random fluctuations. Its 95% Bootstrap confidence interval ([2.4068, 2.5345] m) shows complete separation without overlap compared to other algorithms, further verifying the high robustness and reliability of this method. Furthermore, the maximum single-epoch runtime among all eight algorithms is only 17.8252 ms (L1-LP), fully meeting the engineering requirements for 1 Hz real-time processing.

As demonstrated by the statistical results of Scenario 1 and Scenario 2, the proposed L1-WRM exhibits superior positioning accuracy, robustness, and statistical significance advantages across two distinct degraded environments. In Scenario 1, the 3D Mean Error of L1-WRM is reduced to 2.0190 m, outperforming standard L1-norm algorithms and the traditional robust algorithm MWLS. In Scenario 2, L1-WRM effectively suppresses the influence of outliers in pseudorange observations, maintaining a 3D Mean Error of 2.4643 m, which again outperforms the traditional robust algorithm MWLS. Non-parametric hypothesis testing further confirms the statistical validity of the performance improvement: the global Kruskal–Wallis test *p*-values for both Scenario 1 and Scenario 2 are less than 0.001. Post hoc pairwise Wilcoxon signed-rank tests indicate that L1-WRM significantly outperforms all baseline algorithms in both scenarios. Regarding spatial distribution, the 95% Bootstrap confidence intervals of L1-WRM ([1.9833, 2.0549] m and [2.4068, 2.5345] m, respectively) maintain a complete separation without overlap compared to all other algorithms, fully verifying the high reliability and stability of the estimation algorithm. Additionally, the single-epoch runtimes of all five algorithms based on the MAMEMs meet the engineering requirements for 1 Hz real-time processing.

Scenarios 3 and 4 do not contain multipath or NLOS effects, serving as a contrast to Scenarios 1 and 2. The statistical accuracy results for these two scenarios are shown in Table 6, Table 7, Table 8 and Table 9; the number of combinations for L1-Gauss is 1771 in Scenario 3 and 364 in Scenario 4.

As can be seen from Table 6 and Table 7, in Scenario 3 (without the impact of multipath or NLOS effects), non-parametric hypothesis testing indicates that performance differences among the algorithms remain statistically significant (p≪0.001). However, from the perspective of practical physical accuracy, the positioning performance of all algorithms is extremely close: WLS achieves the lowest 3D Mean Error across the scenario (1.6415 m), whereas the proposed L1-WRM yields 1.7117 m, representing a physical accuracy difference of only 7.02 cm. This minor difference aligns with adjustment theory—the robust weight mapping mechanism sacrifices a minimal amount of asymptotic statistical efficiency under ideal conditions in exchange for immunity against unknown outliers. The 95% Bootstrap confidence intervals of all algorithms show significant overlap, and the maximum single-epoch runtime is 20.3625 ms, fully meeting the requirements for 1 Hz real-time processing.

As can be seen from Table 8 and Table 9, in Scenario 4 (without the impact of multipath or NLOS effects), non-parametric hypothesis testing demonstrates that performance differences among algorithms remain statistically significant p≪0.001). Non-robust algorithms (LS, WLS, bcWLS) achieve optimal estimation under this scenario, with 3D Mean Errors of 0.7698 m, 0.7903 m, and 0.7707 m, respectively. The proposed L1-WRM algorithm yields a 3D Mean Error of 0.8476 m, suffering a slight degradation compared to non-robust algorithms, yet achieving the highest positioning accuracy among all robust-type algorithms. The 95% Bootstrap confidence intervals of all algorithms are extremely narrow, proving that each algorithm possesses high numerical stability in this environment. Regarding the maximum single-epoch runtime, L1-WRM exhibits the longest time at 12.6614 ms, satisfying high-frequency real-time processing requirements of 1 Hz or above. Comparing Scenario 3 and Scenario 4 reveals that in the absence of multipath or NLOS effects, the performance differences among algorithms show no obvious relationship with baseline length.

Combining the above experimental results, under scenarios affected by multipath or NLOS effects, the non-robust algorithm bcWLS outperforms the WLS algorithm, and the robust algorithm L1-WRM outperforms MWLS. Under scenarios free from multipath or NLOS effects, robust algorithms sacrifice partial statistical efficiency in exchange for resistance against outliers. In this case, non-robust algorithms (LS, WLS, bcWLS) outperform the five robust algorithms (L1-Gauss, L1-LP, L1-RM, L1-WRM, MWLS). The positioning accuracy of all eight algorithms across the four scenarios exhibits statistical significance, and the maximum single-epoch runtime is 95.5939 ms, fully meeting the requirements for 1 Hz real-time processing.

## 5. Conclusions

Observational data from sources such as GNSS, EDM, VLBI, and LiDAR have been proven to be susceptible to mixed additive and multiplicative errors. Current research on MAMEMs is predominantly based on the least squares criterion and simulated data. Compared with least squares, L1-norm estimation exhibits superior robustness. To date, however, little research has been conducted on L1-norm estimation specifically for the MAMEMs. In view of this, this study proposes four L1-norm estimation algorithms for the MAMEMs, namely L1-Gauss, L1-LP, L1-RM, and L1-WRM. The performance of these methods was compared against bcWLS and AEM algorithms (LS, WLS and MWLS) using real-world GNSS data. The following conclusions were drawn:

(i) In Scenarios 1 and 2, which are affected by multipath or NLOS effects, the non-robust algorithm bcWLS under the MAMEMs outperforms the non-robust algorithm WLS under the AEMs. Its 3D Mean Errors are 2.6344 m and 5.4061 m, with 95% Bootstrap CIs of [2.5702, 2.7064] m and [5.3063, 5.5192] m, respectively. The robust algorithm L1-WRM under the MAMEMs outperforms the robust algorithm MWLS under the AEMs, with 3D Mean Errors of 2.0190 m and 2.4643 m, and 95% Bootstrap CIs of [1.9833, 2.0549] m and [2.4068, 2.5345] m, respectively.
(ii) In Scenarios 3 and 4, which are free from multipath or NLOS effects, the accuracy of the non-robust algorithm bcWLS under the MAMEMs is basically consistent with that of the non-robust algorithm WLS under the AEMs, with a difference in 3D Mean Error of only around 2 cm. The accuracy of the robust algorithm L1-WRM under the MAMEMs remains basically consistent with that of the robust algorithm MWLS under the AEMs, with a difference in 3D Mean Error of only about 8 cm. Overall, non-robust algorithms exhibit better accuracy than robust algorithms in these scenarios.
(iii) Regarding single-epoch runtime, the maximum value among the five MAMEMs algorithms proposed in this paper is 95.5939 ms, fully satisfying the requirements for 1 Hz real-time processing.

## Figures and Tables

**Figure 1:**
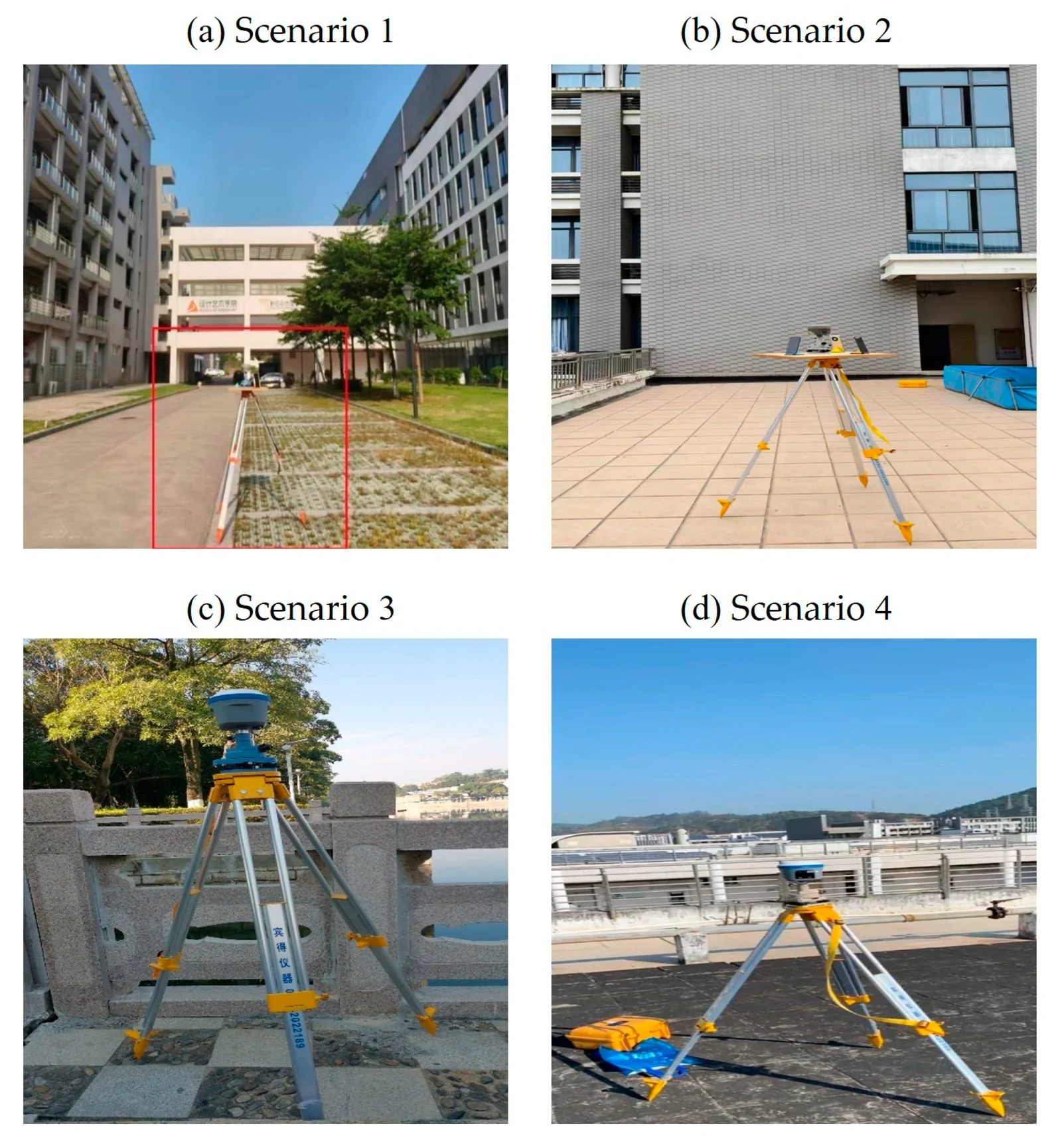
Rover location.

**Figure 2:**
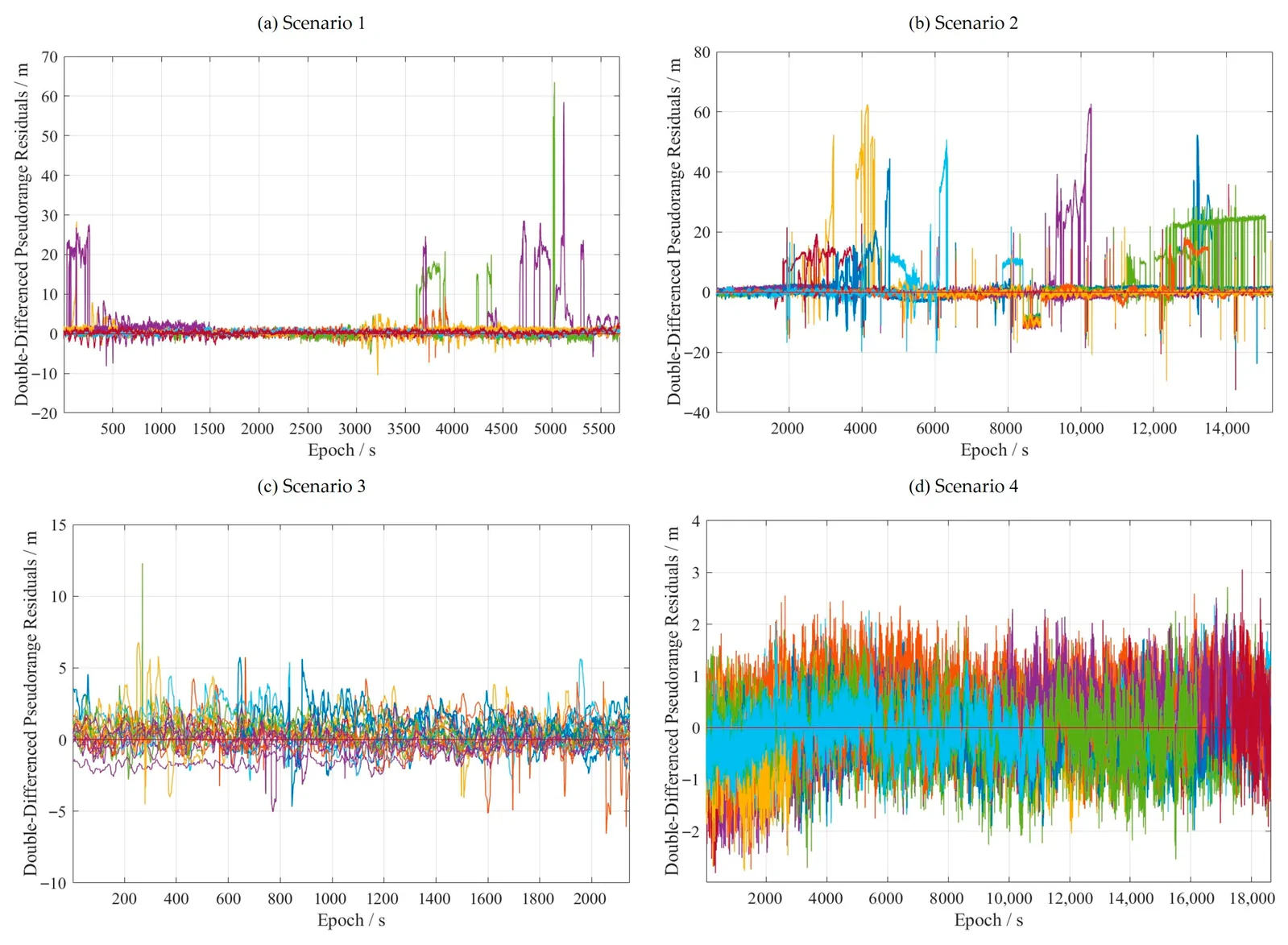
Double-differenced pseudorange residuals in four scenarios.

**Table 1:** Abbreviations and their corresponding definitions.

Abbreviation	Full Name
LS	Standard Least Squares
WLS	Weighted Least Squares
MWLS	M-robust Weighted Least Squares
bcWLS	bias-corrected Weighted Least Squares
L1-Gauss	L1-norm-based Gauss Theorem Algorithm
L1-LP	L1-norm-based Linear Programming Algorithm
L1-RM	L1-norm-based Relaxation Method
L1-WRM	L1-norm-based Weighted Relaxation Method

**Table 2:** RMSE of eight algorithms in Scenario 1 (m).

N-E-U	Algorithms							
	LS	WLS	MWLS	bcWLS	L1-Gauss	L1-LP	L1-RM	L1-WRM
N	4.2184	3.9554	2.2404	1.5565	1.6875	1.6875	1.6899	0.9849
E	1.1980	1.1123	0.9608	1.0299	0.7675	0.7675	0.7675	0.6404
U	4.7973	4.7976	3.9243	3.1282	2.4711	2.4711	2.4626	2.1283

**Table 3:** Significance test results and maximum single-epoch runtime of eight algorithms in Scenario 1.

Algorithms	3D Mean Error (m)	95% Bootstrap CI (m)	vs. L1-WRM (*p*)	Max (ms)
LS	3.9952	[3.8653, 4.1337]	≪0.001	0.5047
WLS	3.9239	[3.8051, 4.0566]	≪0.001	0.1375
MWLS	2.8467	[2.7529, 2.9441]	≪0.001	5.6595
bcWLS	2.6344	[2.5702, 2.7064]	≪0.001	23.7149
L1-Gauss	2.2958	[2.2476, 2.3608]	≪0.001	13.8163
L1-LP	2.2958	[2.2457, 2.3549]	≪0.001	95.5939
L1-RM	2.2850	[2.2328, 2.3375]	≪0.001	3.2250
L1-WRM	2.0190	[1.9833, 2.0549]	Ref.	12.1403

Note: Kruskal–Wallis overall test p≪0.001.

**Table 4:** RMSE of eight algorithms in Scenario 2 (m).

N-E-U	Algorithms							
	LS	WLS	MWLS	bcWLS	L1-Gauss	L1-LP	L1-RM	L1-WRM
N	4.1806	3.6892	2.3590	2.6962	2.7363	2.7363	2.7282	1.9370
E	2.4801	2.1052	1.0233	1.5619	1.4172	1.4172	1.4031	0.8911
U	13.4635	12.5654	6.0169	8.0556	8.4441	8.4441	8.4579	4.0969

**Table 5:** Significance test results and maximum single-epoch runtime of eight algorithms in Scenario 2.

Algorithms	3D Mean Error (m)	95% Bootstrap CI (m)	vs. L1-WRM (*p*)	Max (ms)
LS	8.8652	[8.6812, 9.0342]	≪0.001	0.5543
WLS	8.4048	[8.2478, 8.5799]	≪0.001	0.1326
MWLS	2.9847	[2.9000, 3.0842]	≪0.001	12.4840
bcWLS	5.4061	[5.3063, 5.5192]	≪0.001	11.5144
L1-Gauss	3.9306	[3.7966, 4.0513]	≪0.001	5.0884
L1-LP	3.9306	[3.8024, 4.0583]	≪0.001	17.8252
L1-RM	3.9498	[3.8168, 4.0830]	≪0.001	3.4478
L1-WRM	2.4643	[2.4068, 2.5345]	Ref.	14.1703

Note: Kruskal–Wallis overall test p≪0.001.

**Table 6:** RMSE of eight algorithms in Scenario 3 (m).

N-E-U	Algorithms							
	LS	WLS	MWLS	bcWLS	L1-Gauss	L1-LP	L1-RM	L1-WRM
N	0.4402	0.4328	0.5473	0.4330	0.4755	0.4755	0.4699	0.4715
E	1.0606	1.0265	1.0041	1.0683	0.9813	0.9813	0.9820	0.9917
U	1.3110	1.3182	1.5928	1.2982	1.4618	1.4618	1.4511	1.4494

**Table 7:** Significance test results and maximum single-epoch runtime of eight algorithms in Scenario 3.

Algorithms	3D Mean Error (m)	95% Bootstrap CI (m)	vs. L1-WRM (*p*)	Max (ms)
LS	1.6558	[1.6320, 1.6799]	≪0.001	1.1332
WLS	1.6415	[1.6170, 1.6630]	≪0.001	0.1495
MWLS	1.8223	[1.7946, 1.8567]	≪0.001	9.8948
bcWLS	1.6530	[1.6297, 1.6754]	≪0.001	16.0860
L1-Gauss	1.7158	[1.6899, 1.7426]	≪0.001	20.3625
L1-LP	1.7158	[1.6902, 1.7401]	≪0.001	17.1421
L1-RM	1.7084	[1.6820, 1.7327]	≪0.001	3.2914
L1-WRM	1.7117	[1.6868, 1.7406]	Ref.	17.9692

Note: Kruskal–Wallis overall test p≪0.001.

**Table 8:** RMSE of eight algorithms in Scenario 4 (m).

N-E-U	Algorithms							
	LS	WLS	MWLS	bcWLS	L1-Gauss	L1-LP	L1-RM	L1-WRM
N	0.3089	0.2999	0.4082	0.3046	0.3729	0.3729	0.3679	0.3658
E	0.2390	0.2434	0.3427	0.2397	0.3010	0.3010	0.2958	0.2938
U	0.8052	0.8309	0.9147	0.8046	0.8627	0.8627	0.8550	0.8566

**Table 9:** Significance test results and maximum single-epoch runtime of eight algorithms in Scenario 4.

Algorithms	3D Mean Error (m)	95% Bootstrap CI (m)	vs. L1-WRM (*p*)	Max (ms)
LS	0.7698	[0.7630, 0.7762]	≪0.001	0.5129
WLS	0.7903	[0.7844, 0.7971]	≪0.001	0.0986
MWLS	0.9189	[0.9116, 0.9266]	≪0.001	11.9863
bcWLS	0.7707	[0.7645, 0.7770]	≪0.001	11.5260
L1-Gauss	0.8596	[0.8532, 0.8669]	≪0.001	10.1717
L1-LP	0.8596	[0.8528, 0.8667]	≪0.001	10.6743
L1-RM	0.8502	[0.8435, 0.8566]	≪0.001	3.8532
L1-WRM	0.8476	[0.8409, 0.8546]	Ref.	12.6614

Note: Kruskal–Wallis overall test p≪0.001.

## Data Availability

Some or all data, models, or code generated or used during the study are available from the corresponding author by reasonable request.
